# ASO Author Reflections: Do Surgeon Practice Patterns Follow National Guidelines for Axillary Staging?

**DOI:** 10.1245/s10434-021-10601-1

**Published:** 2021-08-12

**Authors:** Ava Armani, Sasha Douglas, Swati Kulkarni, Anne Wallace, Sarah Blair

**Affiliations:** 1 Department of Surgery, University of California-San Diego, San Diego, CA USA; 2 Department of Surgery, Northwestern University, Chicago, IL USA

## Past

Sentinel lymph node biopsy (SLNB) has been the standard of care for clinically node-negative women with invasive breast cancer (IBC) and is also performed for women with ductal carcinoma in situ (DCIS) with high-risk features undergoing breast conservation surgery (BCS). Despite national guidelines, there is still controversy on whether to perform SLNB when the risk of metastasis is low or when it does not affect survival or locoregional control.

## Present

National guidelines indicate that SLNB should not be routinely performed in women over 70 years of age with early-stage hormone receptor-positive (HR+) Her2 negative IBC^1^ on the basis of studies showing that axillary staging has no impact on regional control or survival^2^ and results in improved early quality of life.^3^ This study^4^ surveyed active members of the American Society of Breast Surgeons (ASBrS) and found that 83% recommend SLNB for a healthy 75-year-old woman with early-stage HR+ Her2 negative IBC despite these recommendations. Surgeons in academic settings were more likely to omit SLNB in this scenario. Almost half of respondents indicated that multidisciplinary teams encourage them to perform SLNB.

Similarly, one-third of ASBrS surgeons surveyed recommend SLNB for DCIS with high-risk features when guidelines advocate against it. Surgeons in academic settings were less likely to perform SLNB in this setting.

## Future

Despite national guidelines, most surgeons favor SLNB in older patients with early-stage HR+ Her2 negative IBC. Clinical factors such as tumor grade, stage, and histology may be used to predict nodal positivity in this population to tailor the omission of SLNB to the subset with low-risk features.^5^ For DCIS undergoing BCS, one-third recommend SLNB. Academic surgeons were more likely to be practicing based on recent data. Better methods of dissemination, education, and de-implementation strategies could help decrease overtreatment in patients that do not benefit from axillary staging. In addition, respondents were greatly influenced by multidisciplinary teams, suggesting that putting forth guidelines across specialties could also improve physician adherence.
